# The Role of Genetic Factors in Characterizing Extra-Intestinal Manifestations in Crohn’s Disease Patients: Are Bayesian Machine Learning Methods Improving Outcome Predictions?

**DOI:** 10.3390/jcm8060865

**Published:** 2019-06-17

**Authors:** Daniele Bottigliengo, Paola Berchialla, Corrado Lanera, Danila Azzolina, Giulia Lorenzoni, Matteo Martinato, Daniela Giachino, Ileana Baldi, Dario Gregori

**Affiliations:** 1 Unit of Biostatistics, Epidemiology and Public Health, Department of Cardiac, Thoracic, and Vascular Sciences and Public Health, University of Padova, 35131 Padova, Italy; daniele.bottigliengo@phd.unipd.it (D.B.); corrado.lanera@unipd.it (C.L.); danila.azzolina@unipd.it (D.A.); giulia.lorenzoni@unipd.it (G.L.); matteo.martinato@unipd.it (M.M.); ileana.baldi@unipd.it (I.B.); 2 Department of Clinical and Biological Sciences, University of Torino, 10126 Torino, Italy; paola.berchialla@unito.it (P.B.); daniela.giachino@unito.it (D.G.)

**Keywords:** Crohn’s disease, extra-intestinal manifestation, risk prediction, Bayesian methods, machine learning techniques

## Abstract

(1) Background: The high heterogeneity of inflammatory bowel disease (IBD) makes the study of this condition challenging. In subjects affected by Crohn’s disease (CD), extra-intestinal manifestations (EIMs) have a remarkable potential impact on health status. Increasing numbers of patient characteristics and the small size of analyzed samples make EIMs prediction very difficult. Under such constraints, Bayesian machine learning techniques (BMLTs) have been proposed as a robust alternative to classical models for outcome prediction. This study aims to determine whether BMLT could improve EIM prediction and statistical support for the decision-making process of clinicians. (2) Methods: Three of the most popular BMLTs were employed in this study: Naϊve Bayes (NB), Bayesian Network (BN) and Bayesian Additive Regression Trees (BART). They were applied to a retrospective observational Italian study of IBD genetics. (3) Results: The performance of the model is strongly affected by the features of the dataset, and BMLTs poorly classify EIM appearance. (4) Conclusions: This study shows that BMLTs perform worse than expected in classifying the presence of EIMs compared to classical statistical tools in a context where mixed genetic and clinical data are available but relevant data are also missing, as often occurs in clinical practice.

## 1. Introduction

Inflammatory bowel disease (IBD) can have various clinical presentations, making the classification of patients very challenging. In subjects affected by Crohn’s disease (CD), extra-intestinal manifestations (EIMs) significantly impact health status. Several European studies report EIMs are present in 20–40% of Crohn’s disease (CD) patients, are more common in females than males and are associated with a duration of disease >10 years (48.9% vs. 29.9% in patients with diseases <10 years) [1,2,3]. Joint manifestations (peripheral or axial arthropathies) are the most common EIMs in IBD and occur in 20–30% of patients, with symptoms ranging from noninflammatory arthralgia to acute arthritis with painful swollen joints, usually associated with active IBD [4]. Articular manifestations may be more frequent in CD patients with stenosing/penetrating disease [4]. Erythema nodosum, pyoderma gangrenosum, and aphthous stomatitis are the most common cutaneous manifestations in IBD. Prevalence rates are 5–15% in CD patients with female predominance, and they parallel disease activity in up to 92% of episodes and may recur in approximately 20–30% of patients [5]. The most common ocular manifestations include uveitis and episcleritis. Their prevalence is between 3% and 6% in CD, and they are associated with disease activity in up to 78% of episodes, with recurrent episodes in approximately 30% of patients [5]. Ocular manifestations may frequently occur together with other (joint or cutaneous) EIMs [6]. Primary sclerosing cholangitis (PSC) may be present in 0.7–2% patients with CD; however, if small-duct PSC is included, the overall prevalence ranges from 2.4 to 11% [4,7]. 

Many strategies have been proposed to appraise the role played by genetic factors in the risk of EIMs occurrence [8]. Knowledge of the risk of EIMs associated with a patient’s characteristics is important for better planning and more tailored treatment strategies [9]. Nevertheless, the relationship between the genetic characteristics of the patient and the occurrence of EIMs remains unclear [10].

Predicting the occurrence of EIMs on the basis of a patient’s characteristics is a challenging task for two main reasons: there is an increasing amount of information on subjects’ characteristics and a limited number of patients for whom data are available [11,12,13]. The high complexity of EIM prediction requires more powerful and complex statistical methods that provide valuable information from a clinical point of view. Indeed, traditional statistical methods, such as simple/multivariate logistic regression, may not represent the best solutions to characterize such a complex risk structure, which may often be characterized by nonlinear relationships between outcome and predictors and interactions among covariates.

Traditional Machine Learning Techniques (MLTs) have been promoted as a promising approach for modeling the role of genetic factors in EIM prediction [14]. The integration of the Bayesian frameworks in the MLTs field has been recently proposed and the use of Bayesian machine learning techniques (BMLTs) is rapidly becoming popular in the medical setting. The ability of these methods to embrace the modeling flexibility of MLTs [15,16,17] with the advantages of Bayesian inference makes them potentially robust in situations in which standard methods may fail, such as handling missing data, robustness to overfitting in presence of small sample size and embedding of external information [18,19].

Some of the most used BMLTs in the medical field are Naϊve Bayes (NB) and Bayesian Network (BN) [20,21,22,23]. Additionally, in the last few years, Bayesian Additive Regression Trees (BART) have been emerging as a frequent choice [24,25]. 

The goal of this study is to understand whether the prediction of EIMs using patients’ clinical and genetic characteristics is enhanced by using novel and existing BMLT approaches. Moreover, we aim to compare the predictive performances of these methods to provide suggestions and support in terms of implementation of modeling strategies.

## 2. Material and Methods

The dataset employed in this study is the same set used in Giachino et al. [14]. It refers to an observational study on the role of genetic factors for IBD. Information on subjects with CD and ulcerative colitis (UC) was retrieved in cooperation with three gastroenterology units in Torino, Italy. Overall, 152 subjects were enrolled in the study. Clinical and familial information on patients was acquired and labeled following the Vienna classification employed at the time of the study. EIMs were defined as manifestations of rheumatological, dermatological, ocular, liver, biliary, and amyloidosis symptoms. Patient characteristics that could potentially affect EIM occurrence were retrieved and divided into two main groups: (i) characteristics of the disease and factors that are known to be associated with the clinical endpoint—age at onset (age), location (location), behavior (behavior), presentation of the disease (onset), gender (gender), smoker status (smoker), family history (history); and (ii) genetic polymorphisms of the following genes: *NOD2, CD14, TNF, IL12B* and *IL1RN*. 

According to the Vienna classification, patients are defined as A1 if the diagnosis is made before 40 years of age and A2 if it was made after 40 years of age. Regarding location, L1 corresponds to disease located in the terminal ileum and possibly involving the caecum, L2 corresponds to colonic disease, L3 to ileocolonic disease, and L4 to CD involving the upper gastrointestinal tract (irrespective of the other locations of the disease). Regarding behavior, B1 corresponds to a non-structured and nonpenetrating disease, B2 to a structured disease, and B3 to a penetrating disease.

Presentation of the disease (onset) refers to medical when a medical diagnosis related to signs and symptoms that provided a suggestion of Crohn’s disease to a medical doctor was made without previous bowel surgery. Surgical refers to cases when the first event of CD was surgery; i.e., a patient was admitted to a hospital for a surgical indication, and a diagnosis of Crohn’s disease was made during the surgical operation or was based on histological analysis of the removed bowel. Family history refers to having a relative with CD (the strongest risk factor for CD, i.e., first-degree relatives of patients with CD have a 12- to 15-fold greater risk of developing CD than do people of comparable age in the general population).

### 2.1. BMLTs

NB, BN, and BART are marked by high flexibility and good prediction ability, which make them very popular among BMLTs and widely applied in the medical field [26]. 

The NB classifier can be represented as a simple Directed Acyclic Graph (DAG), and is very easy to build. In graphical notation, each variable is represented by a node, and the relationships between variables are described by arcs with edges from node to node. The simplicity of NB lies in its naïve assumptions, i.e., the outcome variable is considered the parent node of all other nodes, which are also called child nodes, and there is no connection between child nodes [27]. The fixed structure of this graph makes its implementation very simple. Once the structure of the graph is built, classification is carried out using Bayes’ theorem by computing the posterior predictive distribution of the probability of observing the outcome of interest given the posterior distributions of the parameters. In the literature, the NB classifier has been shown to perform well [28], especially with small sample size datasets [29].

BNs are DAGs [30] that model the joint probability distribution of a set of variables. The graphical structure of a BN can be implemented by imposing the relationships between nodes with expert opinions on the phenomenon under study or by defining the connections between the variables with learning algorithms. Bayes’ theorem is then used to compute the conditional probabilities of the parameters of the models given the values of the variables, which quantify the relationships among nodes connected. Like NBs, BNs provide information on the probability that an event of interest will occur, given the values of the nodes to which the outcome node is connected. From a clinical point of view, this can be very helpful for a researcher to profile patients given the predicted event risks associated with several characteristics.

BART was introduced by Chipman et al. (2010) [31]. It belongs to the popular family of “ensemble-of-trees” methods, such as Bagging, Random Forest, and Boosting. BART is essentially a sum-of-trees model: it describes the relationship between an outcome of interest and a set of covariates as a sum of many regression or classification trees plus a random component. The contribution of each tree to the total sum is weakened by imposing regularizing prior distributions on the parameters that control the sum-of-trees. Regularizing priors are then able to prevent the contribution of a single tree from dominating the total sum, avoiding the problem of overfitting. BART is then able to provide information on the probability of occurrence of an event of interest given subject characteristics by flexibly relating the clinical endpoint to the potential explanatory variables.

In summary, NB and BN are both graphical models. The former imposes a fixed structure on the network and, despite its strong unrealistic assumptions, it has performed very well in several situations [32]. The latter learns the structure of the network using several algorithms or by relying on expert opinions, which makes its classification ability highly dependent on the learning procedure. Several studies compared NB and BN, showing that both techniques have similar performance [33,34,35]. Moreover, many studies compared the performance of NB and BN with other classical MLTs, showing that they have similar predictive performances with respect to the other classical methods [36,37,38,39,40]. BART belongs to the family of ensemble-of-trees models, and its regularizing prior distributions and ability to put together weak classifiers avoid overfitting. It is a good alternative to other more popular MLTs, such as lasso regression, random forest, and neural networks [31].

### 2.2. Statistical Analysis

Patients were classified according to the presence or absence of EIMs if the predicted probability of the event was higher than 0.5. 

Missing data were inferred with the same strategies used in the study of Giachino et al. [14]: the median of available sample values for continuous variables and an additional level indicating the presence of missingness for categorical variables.

BMLTs were compared with the statistical approaches employed in the study of Giachino et al. [14], i.e., logistic regression (LR), generalized additive model (GAM), projection pursuit regression (PPR), linear discriminant analysis (LDA), quadratic discriminant analysis (QDA), and artificial neural networks (ANN). Statistical analysis was implemented following the same model validation approach adopted in the study mentioned above to perform a fair comparison. Parameters of the models were chosen using repeated *k*-fold cross-validation, setting the number of folds equal to 10 and repeating the process 10 times. Regarding BART models, the Gibbs sampler was used to draw from the posterior distribution using 2000 draws and discarding the first 500 as “burn-in” draws. Convergence of the posterior distribution was assessed by inspecting the acceptance percentage of MCMC proposals across the trees, the average number of leaves, and tree depth at each MCMC iteration after the “burn-in” draws. All of the patients in the sample were considered in the training set. Since no external data for testing the models were available, model validation was carried out using 1000 bootstrapped samples of the original dataset [41], a procedure that has been shown to give unbiased estimates of the error rate [42]. The ability to correctly classify the presence or absence of EIMs was evaluated by comparing several indicators: Somers’ Dxy (Somers’ D), positive and negative predicted values of model predictions (PPV and NPV, respectively), overall misclassification error (MCR, which corresponds to the percentage of observations wrongly classified) and the area under the ROC curve (AUC). Moreover, the sensitivity and specificity of BMLTs were also assessed to provide more information on their predictive ability. We did not use the discrimination index—defined as the likelihood ratio divided by the sample size [42]—to compare the techniques as in Giachino et al. [14]. Rather, following Gelman and Rubin [43], we preferred to explore their posterior distributions by means of their ability to correctly classify patients according to the indicators mentioned above.

For each model, two analyses were performed: one including only the first group of potential explanatory variables as covariates, and one also including genetic variables. Such a strategy was conducted to evaluate the impact that genetic factors have on the overall ability of the models to predict the presence or absence of EIMs.

Regarding the analysis with BN, two elucidations needed to be done. First, several algorithms can be used to learn the structure of the network. The choice of the algorithm is nontrivial, and currently, no rigorous method covers this issue. Since the definition of a procedure for choosing the best algorithm is beyond the scope of this study, the network that achieved the lowest MCR was selected. Second, to implement a more robust model, the structure of the network was chosen with a model averaging approach for each algorithm [44]. The structural learning process was repeated on 1000 bootstrap replicates of the original sample, and the structure of the network was created according to the approach in Scutari and Nagarajan (2013) [45].

All analyses were performed using version 3.4.3 of R software [46] on a HP ProDesk 490 G3 MT Business PC with a Inter(R) Core(TM) i7-6700 CPU @ 3.40GHz processor. NB and BN were implemented using the “bnlearn” package [47], while BART was implemented using the “bartMachine” package [48]. Default prior distributions of the “bnlearn” package were used for NBs and BNs. The data that support the findings of this study are available from the corresponding author upon request. The R code to simulate the data and reproduce the analysis is available on Github (https://github.com/UBESP-DCTV/ibd-bmlt).

## 3. Results

The distributions of the patient’s characteristics by absence/presence of EIMs are reported in Table 1. Overall, 75 subjects (nearly 49%) had EIMs, whereas 77 patients (nearly 51%) did not have EIMs. Onset, behavior, location, age, family history, and polymorphism of *NOD2* and *CD14* showed similar distributions across patients with and without EIMs, whereas the distributions of the other variables were more unbalanced.

MCRs of the BNs learned with different algorithms are reported in Table 2. All the available algorithms in the “bnlearn” package were used to learn the structure of the network. Among the models without genetic variables, the network learned with the SI-HITON-PC algorithm showed the lowest MCR, whereas the poorest performance was observed for the networks learned with the IAMB, Fast-IAMB, and TS algorithms. Among the models with the genetic variables, the best performances were observed for the networks learned with the TS and the HC algorithms, whereas the network learned with the IAMB algorithm showed the highest MCR.

Performance indexes are reported in Table 3. MCRs of LR, GAM, PPR, LDA, QDA, and ANN are taken from the study of Giachino et al. [14].

Regarding models without genetic variables, NB performed similarly to the six techniques employed in the previous study, showing a slightly lower MCR value (0.34), higher NPV value (0.65), and lower PPV value (0.68). A sensitivity and a specificity of 0.45 and 0.81, respectively, were observed. The BN chosen for analysis was the one learned with the Hiton Parents and Children (SI-HITON-PC) algorithm, which was the network with the lowest MCR. The performance was very poor, as suggested by the performance indicator values (MCR = 0.50, PPV = 0.51, NPV = 0.49, AUC = 0.50), and it had practically no ability to correctly discriminate between the presence and absence of EIMs (specificity = 0.00 and Somer’s D = 0.00). BART showed slightly better values for performance indicators than NB and BN (MCR = 0.32, sensitivity = 0.64, specificity = 0.68, PPV = 0.67, NPV = 0.69, AUC = 0.76, Somer’s D = 0.51), but no consistent improvement was achieved concerning the statistical methods previously employed.

When considering genetic variables, NB slightly improved the performance, with higher indicator values compared to those achieved without genetic variables. With respect to the techniques in Giachino et al. [14], it showed a similar MCR value (MCR = 0.33), lower PPV, AUC and Somer’s D values (PPV = 0.69, AUC = 0.75, Somer’s D = 0.51) and a higher NPV value (NPV = 0.66). Sensitivity and specificity were equal to 0.65 and 0.69, respectively. Among the BNs, the network learned with Tabu-Search (TS) and Hill-Climbing (HC) algorithms showed the same structure achieving the lowest MCRs. For comparison purposes, the network learned by TS was selected. Predictive performance was not satisfactory: despite higher values with respect to the BN without genetic variables, BN performance metrics were still lower than those reported in Giachino et al. [14] (PPV = 0.68, NPV = 0.65, AUC = 0.67, Somer’s D = 0.33); however, MCR showed a similar value (0.34). A sensitivity and a specificity of 0.64 and 0.69, respectively, were observed. Like NB, BART improved its performances when also considering genetic factors. With respect to the previous results in Giachino et al. [14], it showed similar MCR, AUC and Somer’s D values, a lower PPV value and a higher NPV value (MCR = 0.32, sensitivity = 0.66, specificity = 0.69, PPV = 0.67, NPV = 0.69, AUC = 0.78, Somer’s D = 0.56). Convergence of the posterior distribution was reached for both BART models: the acceptance percentage of MCMC proposals across the trees, the average number of leaves and tree depth at each MCMC iteration after the “burn-in” period showed stationary processes. 

The analyses with NB and BN took less than 2 min. The analysis carried out with BART was computationally expensive and took approximately 7 h.

## 4. Discussion

In the current study, BMLTs did not show any major improvements in classification accuracy compared to the methods reported in Giachino et al. [14]. In many scenarios, especially when genetic information was also considered, they performed worse. Models with genetic variables showed better performance metrics than models without genetic information. Nevertheless, the improvement did not seem as strong as that shown in Giachino et al. [14], with the exception of BN.

**Figure 1:**
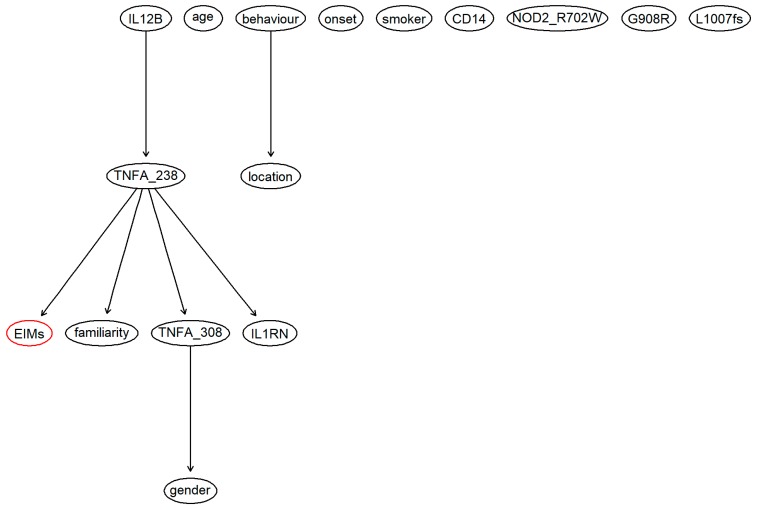
Structure of the Bayesian network estimated considering demographical variables, known risk factors and genetic factors. The red node is the clinical endpoint, which indicates the presence or absence of EIMs. The chosen network was learned with the Tabu Search algorithm, which is one of the available algorithms in the “bnlearn” R package.

Very low performances of BN without genetic factors were observed because the network was not able to associate the node of the clinical endpoint (i.e., the appearance of EIMs) to any other node. Indeed, since the outcome node was not present in the structure of the network, the BN was not able to discriminate between the presence and absence of EIMs, and it classified all of the individuals with a presence of EIMs (EIMs+). By adding genetic factors to the BN, the outcome was included in the network (Figure 1), which allowed it to achieve a higher predictive performance. 

Even though the BN structure with genetic factors was much more articulated and complex than the BN structure without genetic factors, only a few connections between the variables were found in both networks. The structure of the networks learned with all of the other algorithms was further investigated for both scenarios with and without genetic factors. Regarding BN without genetic factors, most of the networks showed an identical structure (the same structure of the network used in the analysis). Even with a more complicated structure, the node of the clinical endpoint was not included in the structure. Adding genetic factors, only networks learned with TS and HC algorithms showed some degree of complexity in their structures, which also included the outcome node.

**Figure 2:**
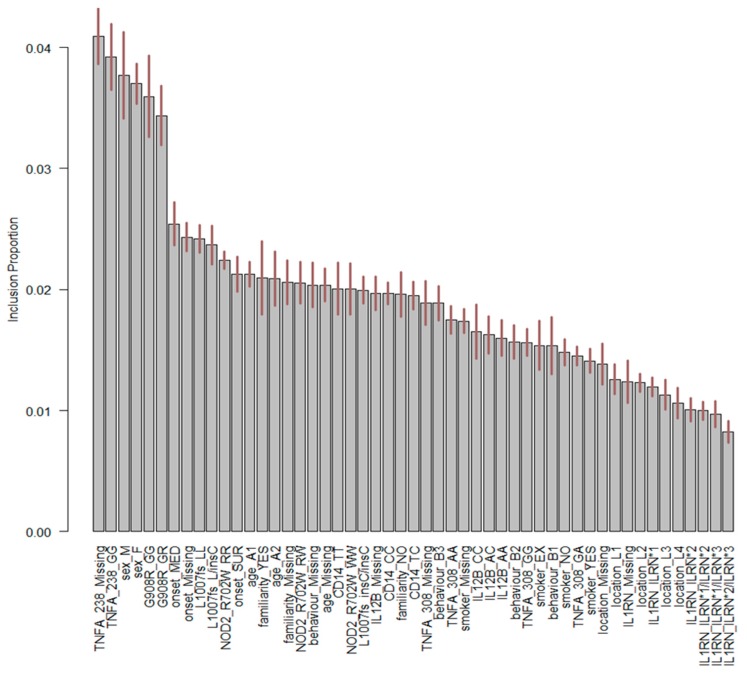
Important variables of BART with genetic factors. On the x-axis, the explanatory variables and their associated levels are shown. The first part of the label refers to the name of the explanatory variable, whereas the second part refers to the label of the classification. Variable labels are reported in Table 1. On the y-axis, the percentage of times for each variable was used to determine a splitting rule.

The predictive performances of BMLTs were investigated by looking at the individual predicted probabilities of the presence of EIMs assigned by each model averaged across every bootstrap sample (Table 4). Two interesting findings were observed. First, all BMLTs predicted individual probabilities of the presence of EIMs less than 0.5 from the first patient to the 72nd patient. From the 73rd patient to the last patient, all BMLTs assigned individual probabilities over 0.5. This was probably because after the 73rd patient, all of the information for the genes *IL12B*, *TNFA-308*, *TNFA-238*, and *IL1RN* was systematically missing, indicating that this portion of missing values could be considered as missing not at random (MNAR). This missing pattern likely affected the performance of the models: patients with missing information for the genes *IL12B*, *TNFA-308*, *TNFA-238*, and *IL1RN* were classified as having EIMs, since a probability higher than 0.5 was assigned to them, whereas the opposite predictive pattern was observed for patients without missing information for the genes. Indeed, looking at Figure 2, *IL12B* and *TNFA-238*, two of the four variables that were MNAR, played a crucial role in the BN structure and therefore in predicting the presence of EIMs. Figure 2 displays BART variable importance, indicating the proportion of times a variable is considered in the definition of the splitting rule on each of the trees constructed by the model. The “Missing” category of gene *TNFA-238* played an important role in the definition of the model.

From a clinical perspective, the relationship between *TNFA-308* and gender depicted in Figure 2 raises some issues since it is somewhat counterintuitive. The unexpected link between TNF-α single nucleotide polymorphism (‒308G>A, rs1800629) and gender may be somehow explained by the increased prevalence of association between ADA (22G>A) and TNF-α (‒308G>A) polymorphisms in Italian males investigated by Napolioni and Predazzi [49].

### 4.1. Study Limitations

Systematic missing entries for the *IL12B*, *TNFA-308*, *TNFA-238*, and *IL1RN* genes in some patients highly affected the performance of the models. 

Moreover, the predictive posterior risk of EIMs was dichotomized to identify the presence of EIMs (presence of EIMs if the risk was higher than 0.5) as in the study from Giachino et al. [14] to make the comparison as fair as possible. As noted in many studies [50,51,52,53], the dichotomization of continuous variables results in a loss of information. The potential gains obtained by exploiting the full posterior predictive probability of EIMs will be explored in future research studies.

Finally, the procedure adopted to choose which BN to use during the analysis was quite simplistic, although it represents a default approach in terms of BN tuning. Robust methods that can define the best algorithm in terms of structure learning should be investigated.

### 4.2. Final Remarks

This study shows that emerging BMLTs do not provide a major improvement in correctly classifying the presence of EIMs compared to the classical statistical tools. When genetic variables are considered in the models, they show even lower performances with respect to the classical methods employed in the study from Giachino et al. [14]. The limited sample size of the datasets and absence of an external source of data most likely limited the validation process and the predictive ability of the models. Nevertheless, BMLTs were expected to improve risk prediction in situations where the available amount of data and information is not optimal to build predictive models, as in our study. Our findings did not support the expectations, and some issues may arise around preference for BMLTs over other classical methods.

## Figures and Tables

**Table 1:** Distributions of patient characteristics across the absence or presence of EIMs. EIMs = No indicates absence of EIMs, EIMs = Yes indicates presence of EIMs. n* refers to the number of records without missing entries.

Variable (n*)		EIM = No, *n* (%) *N* = 77	EIM = Yes, *n* (%) *N* = 75	Combined, *n* (%) *N* = 152
**Onset (147)**	Medical	70 (90.9)	63 (84.0)	133 (87.5)
	Surgical	7 (9.1)	7 (9.3)	14 (9.2)
**Behavior (108)**	B1	25 (32.5)	25 (33.3)	50 (32.9)
	B2	15 (19.5)	20 (26.7)	35 (23.0)
	B3	9 (11.7)	14 (18.7)	23 (15.1)
**Location (109)**	L1	14 (18.2)	14 (18.7)	28 (18.4)
	L2	11 (14.3)	14 (18.7)	25 (16.4)
	L3	21 (27.3)	27 (36.0)	48 (31.6)
	L4	4 (5.2)	4 (5.3)	8 (5.3)
**Age (146)**	A1	53 (68.8)	51 (68.0)	104 (68.4)
	A2	21 (27.3)	21 (28.0)	42 (27.6)
**Gender (152)**	M	46 (59.7)	34 (45.3)	80 (52.6)
	F	31 (40.3)	41 (54.7)	72 (47.4)
**Smoker (146)**	No	42 (54.5)	36 (48.0)	78 (51.3)
	Yes	19 (24.7)	26 (34.7)	45 (29.6)
	Ex	11 (14.3)	12 (16.0)	23 (15.1)
**Family History (139)**	No	58 (75.3)	57 (76.0)	115 (75.7)
	Yes	11 (14.3)	13 (17.3)	24 (15.8)
***NOD2:R702W* (152)**	RR	63 (81.8)	64 (85.3)	127 (83.6)
	RW	11 (14.3)	9 (12.0)	20 (13.2)
	WW	3 (3.9)	2 (2.7)	5 (3.3)
***G908R* (152)**	GG	73 (94.8)	67 (89.3)	140(92.1)
	GR	4 (5.2)	8 (10.7)	12 (7.9)
***L1007fs* (152)**	LL	71 (92.2)	65 (86.7)	136 (89.5)
	L/insC	5 (6.5)	8 (10.7)	13 (8.6)
	insC/insC	1 (1.3)	2 (2.7)	3 (2.0)
***CD14* (152)**	CC	20 (26.0)	20 (26.7)	40 (26.3)
	TC	39 (50.6)	36 (48.0)	75 (49.3)
	TT	18 (23.4)	19 (25.3)	37 (24.3)
***TNF-308* (72)**	GG	35 (45.5)	18 (24.0)	53 (34.9)
	GA	9 (11.7)	4 (5.3)	13 (8.6)
	AA	5 (6.5)	1 (1.3)	6 (3.9)
***TNF -238* (72)**	GG	49 (63.6)	23 (30.7)	72 (47.4)
***IL12B* (72)**	AA	17 (22.1)	11 (14.7)	28 (18.4)
	AC	24 (31.2)	10 (13.3)	34 (22.4)
	CC	8 (10.4)	2 (2.7)	10 (6.6)
***IL1RN* (72)**	ILRN*1	29 (37.7)	12 (16.0)	41 (27.0)
	ILRN*1/ILRN*	15 (19.5)	7 (9.3)	22 (14.5)
	ILRN*2	3 (3.9)	3 (4.0)	6 (3.9)
	ILRN*1/ILRN*	1(1.3)	1 (1.3)	2 (1.3)
	ILRN*2/ILRN*	1 (1.3)	0	1 (0.7)

**Table 2:** MCR of the networks learned with different algorithms. The selected algorithms were the ones implemented in the “bnlearn” R package. Performances for networks with and without genetic variables are reported.

Learning algorithm	MCR
**Model without genetic variables**	
Grow-Shrink (GS)	0.57
Incremental Association Markov-Blanket (IAMB)	0.61
Fast Incremental Association Markov-Blanket (Fast-IAMB)	0.61
Interleaved Incremental Association Markov-Blanket (Inter-IAMB)	0.59
Hill-Climbing (HC)	0.57
Tabu-Search (TS)	0.61
Max-Min Hill-Climbing (MMHC)	0.53
Restricted Maximization (RSMAX2)	0.60
Max-Min Parents and Children (MMPC)	0.55
Hiton Parents and Children (SI-HITON-PC)	0.51
Chow‒Liu (CL)	0.56
ARACNE	0.58
**Model with genetic variables**	
Grow-Shrink (GS)	0.57
Incremental Association Markov-Blanket (IAMB)	0.62
Fast Incremental Association Markov-Blanket (Fast-IAMB)	0.61
Interleaved Incremental Association Markov-Blanket (Inter-IAMB)	0.59
Hill-Climbing (HC)	0.34
Tabu-Search (TS)	0.34
Max-Min Hill-Climbing (MMHC)	0.53
Restricted Maximization (RSMAX2)	0.60
Max-Min Parents and Children (MMPC)	0.56
Hiton Parents and Children (SI-HITON-PC)	0.51
Chow‒Liu (CL)	0.57
ARACNE	0.58

**Table 3:** Indicators of classification ability of the model both with and without genetic variables. Values for LR, GAM, PPR, LDA, QDA, and ANN are the same reported in Giachino et al. [14].

	MCR	Sensitivity	Specificity	PPV	NPV	AUC	Somer’s D
**Model without genetic variables**							
**LR**	0.46	_	_	0.77	0.52	0.72	0.45
**GAM**	0.44	_	_	0.81	0.53	0.72	0.45
**PPR**	0.36	_	_	0.98	0.58	0.82	0.64
**LDA**	0.49	_	_	0.98	0.52	0.70	0.40
**QDA**	0.49	_	_	0.72	0.52	0.67	0.34
**ANN**	0.38	_	_	0.94	0.57	0.79	0.58
**NB**	0.34	0.45	0.81	0.68	0.65	0.71	0.42
**BN**	0.50	1.00	0.00	0.51	0.49	0.50	0.00
**BART**	0.32	0.64	0.68	0.67	0.69	0.76	0.51
**Model with genetic variables**							
**LR**	0.39	_	_	0.89	0.56	0.77	0.53
**GAM**	0.37	_	_	0.90	0.57	0.77	0.54
**PPR**	0.30	_	_	0.99	0.62	0.94	0.87
**LDA**	0.38	_	_	0.99	0.57	0.77	0.53
**QDA**	0.22	_	_	0.74	0.52	0.88	0.75
**ANN**	0.33	_	_	0.92	0.60	0.87	0.73
**NB**	0.33	0.65	0.69	0.69	0.66	0.75	0.51
**BN**	0.34	0.64	0.69	0.68	0.65	0.67	0.33
**BART**	0.32	0.66	0.69	0.67	0.69	0.78	0.56

**Table 4:** Average individual predicted probabilities for the presence of EIMs assigned by each model across each bootstrap replicate of the original sample. From the patient with ID = 73 to the last patient, entries for the genetic factors *IL12B*, *TNFA-308*, *TNFA-238*, and *IL1RN* were systematically missing.

ID	EIMs	*IL12B*	*TNFA-308*	*TNFA-238*	*IL1RN*	NB	BN	BART
63	NO	AC	GG	GG	ILRN*1	0.06	0.36	0.26
64	NO	AA	GG	GG	ILRN*1/ILRN*3	0.13	0.36	0.29
65	NO	AC	GG	GG	ILRN*1	0.15	0.36	0.48
66	NO	AA	GG	GG	ILRN*1	0.22	0.36	0.48
67	NO	AC	GG	GG	ILRN*1	0.02	0.36	0.35
68	NO	AC	GG	GG	ILRN*1	0.03	0.36	0.31
69	NO	AA	GA	GG	ILRN*1/ILRN*2	0.09	0.36	0.32
70	NO	AA	AA	GG	ILRN*1	0.04	0.36	0.37
71	YES	AA	GA	GG	ILRN*1	0.02	0.36	0.28
72	YES	AA	GG	GG	ILRN*2	0.28	0.36	0.36
73	YES	Missing	Missing	Missing	Missing	0.91	0.65	0.65
74	NO	Missing	Missing	Missing	Missing	0.69	0.65	0.61
75	YES	Missing	Missing	Missing	Missing	0.98	0.65	0.70
76	YES	Missing	Missing	Missing	Missing	0.95	0.65	0.65
77	YES	Missing	Missing	Missing	Missing	0.97	0.65	0.64
78	YES	Missing	Missing	Missing	Missing	0.95	0.65	0.68
79	YES	Missing	Missing	Missing	Missing	0.98	0.65	0.70
80	YES	Missing	Missing	Missing	Missing	0.88	0.65	0.68
81	YES	Missing	Missing	Missing	Missing	0.93	0.65	0.68
82	YES	Missing	Missing	Missing	Missing	0.98	0.65	0.75
